# Interleukin-1β induces fibroblast growth factor 2 expression and subsequently promotes endothelial progenitor cell angiogenesis in chondrocytes

**DOI:** 10.1042/CS20150622

**Published:** 2016-03-18

**Authors:** Szu-Yu Chien, Chun-Yin Huang, Chun-Hao Tsai, Shih-Wei Wang, Yu-Min Lin, Chih-Hsin Tang

**Affiliations:** * Graduate Institute of Clinical Medical Science, China Medical University, Taichung, Taiwan; † Department of Orthopaedic Surgery, China Medical University Beigang Hospital, Yun-Lin County, Taiwan; ‡ School of Medicine, China Medical University, Taichung, Taiwan; § Department of Orthopedic Surgery, China Medical University Hospital, Taichung, Taiwan; ║ Department of Medicine, Mackay Medical College, New Taipei City, Taiwan; ¶ Institute of Medicine, Chung Shan Medical University, Taichung, Taiwan; ** Department of Orthopedic Surgery, Taichung Veterans General Hospital, Taichung, Taiwan; †† Graduate Institute of Basic Medical Science, China Medical University, Taichung, Taiwan; ‡‡ Department of Biotechnology, College of Health Science, Asia University, Taichung, Taiwan

**Keywords:** arthritis, cartilage, chondrocyte, FGF-2, IL-1β, neovascularization

## Abstract

Arthritis is a process of chronic inflammation that results in joint damage. IL (interleukin)-1β is an inflammatory cytokine that acts as a key mediator of cartilage degradation, and is abundantly expressed in arthritis. Neovascularization is one of the pathological characteristics of arthritis. However, the role of IL-1β in the angiogenesis of chondrocytes remains unknown. In the present study, we demonstrate that stimulating chondrocytes (ATDC5) with IL-1β increased the expression of FGF (fibroblast growth factor)-2, a potent angiogenic inducer, and then promoted EPC (endothelial progenitor cell) tube formation and migration. In addition, FGF-2-neutralizing antibody abolished ATDC5-conditional medium-mediated angiogenesis *in vitro*, as well as its angiogenic effects in the CAM (chick chorioallantoic membrane) assay and Matrigel plug nude mice model *in vivo*. IHC (immunohistochemistry) staining from a CIA (collagen-induced arthritis) mouse model also demonstrates that arthritis increased the expression of IL-1β and FGF-2, as well as EPC homing in articular cartilage. Moreover, IL-1β-induced FGF-2 expression via IL-1RI (type-1 IL-1 receptor), ROS (reactive oxygen species) generation, AMPK (AMP-activated protein kinase), p38 and NF-κB (nuclear factor κB) pathway has been demonstrated. On the basis of these findings, we conclude that IL-1β promotes FGF-2 expression in chondrocytes through the ROS/AMPK/p38/NF-κB signalling pathway and subsequently increases EPC angiogenesis. Therefore IL-1β serves as a link between inflammation and angiogenesis during arthritis.

## Abstract (precis)

Angiogenesis is an important event in the process of arthritis. Stimulating chondrocytes with IL-1β increased the expression of FGF-2, via the IL-1RI/ROS/AMPK/p38/NF-κB signalling pathway. FGF-2-neutralizing antibody abolished ATDC5-conditional medium-mediated angiogenesis both *in vitro* and *in vivo*.

## CLINICAL PERSPECTIVES

• Arthritis is a process of inflammation that results in joint damage. One of the crucial mediators of the inflammatory response is IL-1β. Angiogenesis is now identified as a key event in the process of arthritis and is highly associated with inflammation. However, the role of IL-1β in the angiogenesis of chondrocytes remains unknown.
• In the present study, stimulating ATDC5 with IL-1β increased the expression of FGF-2, a potent angiogenic inducer, and then promoted EPC angiogenesis. In addition, FGF-2-neutralizing antibody abolished ATDC5-conditioned medium-mediated angiogenesis *in vitro*, as well as its angiogenic effects in the CAM assay, Matrigel plug nude mice model and CIA mouse model *in vivo*. Moreover, IL-1β induced FGF-2 expression via IL-1RI, ROS generation, AMPK, p38 and NF-κB pathway.
• The present study provides a better understanding of the mechanisms of angiogenesis underlying arthritis pathogenesis, and opens a new window in the search for potential therapeutic targets of arthritis.

## INTRODUCTION

Arthritis is a process of joint dysfunction, with acute or long-term inflammation that afflicts one or more joints of the patient. The most common forms of arthritis are OA (osteoarthritis) and RA (rheumatoid arthritis). The symptoms of arthritis include swelling, pain, joint stiffness and eroded cartilage [1]. Several factors are associated with an increased risk of arthritis, including aging, obesity, genetic factors and joint injury [2]. In addition, abnormal reactions of the immune system and genetic factors may contribute to arthritis [3]. However, the underlying mechanisms and the pathogenesis of arthritis remain unknown.

There are several cytokines involved in the intra-articular inflammation of arthritis. One of the crucial mediators of the inflammatory response is IL (interleukin)-1β, which is a member of the IL-1 family [4,5]. Recent research has shown that IL-1β is one of the pivotal cytokines in initiating the processes of RA [6,7]. In addition, the concentrations of IL-1β in synovial fluid are elevated in RA patients, and are correlated with various parameters of disease activity [8,9]. Moreover, IL-1 expression is markedly increased in the chondrocytes, synovial cells and synovial fluid of OA patients, with a positive correlation between the expression of IL-1β and the severity of chondral damage [10]. Otherwise, IL-1β is produced in macrophages, synovium, subchondral bone and cartilage, and acts as a key mediator of cartilage degradation [6,11–13] through IL-1RI (type-1 interleukin-1 receptor) [7,14,15].

Pannus and neovascularization of the osteochondral junction are two important characteristics of structure remodelling in arthritis [1,16–18]. Inflammation is intimately linked to angiogenesis; high levels of angiogenic activity in the joint area may promote the procession of arthritis [17,19]. FGF (fibroblast growth factor)-2 is a ubiquitously expressed pleiotropic growth factor of the FGF family, and a powerful inducer of angiogenesis [20,21]. Treatment of endothelial cells with FGF-2 promoted an angiogenic phenotype, with increased cell proliferation, migration and expression of specific integrins [22]. Previous studies also revealed that high FGF-2 levels were found in the synovial fluid of OA and RA patients compared with that of normal individuals [23]. Significant FGF-2 staining in the hyperplastic lining of synoviocytes and at the pannus–cartilage interface of RA synovial tissues suggests that FGF-2 plays a role in the pathology of RA [21,24]. EPC (endothelial progenitor cell) recruitment from the bone marrow to the relevant sites and then differentiating into endothelial cells have been characterized in blood vessel repair and neovascularization [25–27]. In addition, growth factors, such as FGF-2, were found to regulate multiple functions of angiogenesis in EPCs, including proliferation, migration and capillary-like tube formation [25,28].

Healthy adult joint cartilage is an avascular tissue, and FGF-2 is stored within the pericellular matrix of chondrocytes. During arthritis, IL-1β-induced catabolic processes promote matrix degradation and result in the formation of microcracks in the cartilage, which leads to FGF-2 release [19]. Meanwhile, EPCs from the circulatory system migrate and invade into the joint [29,30], facilitating angiogenesis in arthritis. Hence the inhibition of angiogenesis has been proposed as a novel therapeutic strategy for arthritis [31,32]. However, the relationship between IL-1β and angiogenesis in chondrocytes remains unclear. In the present study, we focus on the role of IL-1β in FGF-2 expression and angiogenesis in chondrocytes. We reveal that the activation of the ROS (reactive oxygen species), AMPK (AMP-activated protein kinase), p38 and NF-κB (nuclear factor κB) pathway is required for IL-1β-induced FGF-2 expression and for EPC angiogenesis in chondrocytes.

## MATERIALS AND METHODS

### Materials

HRP (horseradish peroxidase)-conjugated anti-mouse and anti-rabbit IgG rabbit polyclonal antibodies specific for AMPKα1/2, p38α, NF-κB p65 (p65) and phospho-NF-κB p65 (p-p65), and mouse monoclonal antibodies specific for phospho-p38 (p-p38) were purchased from Santa Cruz Biotechnology. Rabbit polyclonal antibody specific for phospho-AMPKα (p-AMPKα) (Thr^172^) was purchased from Cell Signaling Technology. Rabbit polyclonal antibodies specific for FGF-2, CD31 and CD133, and rabbit monoclonal antibodies specific for CD34, were purchased from Abcam. Mouse monoclonal neutralizing antibody against FGF-2 (FGF-2 NAb) was purchased from Millipore. Goat polyclonal antibodies specific for VEGFR2 (vascular endothelial growth factor receptor 2)/KDR (kinase insert domain-containing receptor), and neutralizing antibodies against IL-1RI (IL-1RI NAb) and recombinant human IL-1β were purchased from R&D Systems. SB203580 was purchased from Enzo Life Sciences. ON-TARGETplus SMARTpool duplex siRNA targeting AMPKα1 (PRKAA1), AMPKα2 (PRKAA2), p38 (MAPK14) and ON-TARGETplus non-targeting siRNA (control) were purchased from Dharmacon Research. NF-κB luciferase plasmid was purchased from Stratagene. DPI (diphenyleneiodonium chloride) was purchased from Biomol. NAC (*N*-acetyl-L-cysteine), Ara A (adenine 9-β-D-arabinofuranoside), Compound C (dorsomorphin), PDTC (ammonium pyrrolidinedithiocarbamate), TPCK (*N*-p-tosyl-L-phenylalanine chloromethylketone), bCII (bovine type II collagen), IFA (incomplete Freund's adjuvant), CFA (complete Freund's adjuvant) and all other chemicals were purchased from Sigma–Aldrich.

### ATDC5 cell lines and culture conditions

The chondrocytic cell line murine ATDC5 was provided by Dr Shyh-Ming Kuo (I-Shou University, Kaohsiung, Taiwan; original cells were purchased from Riken Cell Bank, Tsukuba, Japan). ATDC5 is a well-established *in vitro* culture model, and is commonly used for chondrogenic differentiation and endochondral ossification studies [33,34]. Previous studies indicated that ATDC5 cells expressed two chondrocyte markers (type II and X collagen) [35,36]. In the present study, we also confirmed that the ATDC5 cells expressed the mRNA of type II and X collagen (Supplementary Figure S1). The cells were cultured in a 1:1 mixture of DMEM (Dulbecco's modified Eagle's medium) and Ham's F12 medium (Life Technologies) containing 5% (v/v) FBS (Gibco-BRL). Cells were maintained at 37°C in a humidified atmosphere of 5% CO_2_ as described previously [33,37] until an 80% confluent monolayer of cells was ready for experiments.

### Isolation of human circulating EPCs

EPCs were derived from two different healthy donors who gave informed consent before enrolment. Ethical approval was granted by the Institutional Review Board of Mackay Medical College (New Taipei City, Taiwan; reference number P1000002). EPCs were isolated and purified as described previously [38–40]. Briefly, mononuclear cells were isolated from peripheral blood (80 ml) using the Ficoll-Paque™ plus (GE Healthcare) centrifugation method. Then, EPCs were separated from isolated mononuclear cells using the CD34 MicroBead Kit and the MACS™ Cell Separation System (Miltenyi Biotec). CD34-postive EPCs were seeded on 1% gelatin-coated dishes and maintained in MV2 complete medium consisting of basal medium, SupplementMix (both from PromoCell) and 20% non-heat-inactivated defined FBS (HyClone). Characterization of EPCs was confirmed by UEA-1 (*Ulex europaeus* agglutinin-1) lectin binding and surface marker staining of CD31, CD34 and VEGFR2 using a FACSCalibur™ flow cytometer and CellQuest™ software (BD Biosciences) as described previously [39,40]. Circulating EPCs marker-stained with CD133 [26] were also examined by immunofluorescence in our previous study [29].

### EPC migration assay

The EPC migration assay was performed using Transwell inserts (8.0 μm pore size; Costar) in 24-multiwell plates. EPCs (10^4^ cells in 200 μl of medium with 10% FBS) were then seeded into the upper chamber, and 300 μl of ATDC5 CM (culture medium) was placed in the lower chamber. Cells on the upper side of the Transwell membrane were examined and counted under a microscope.

### EPC tube formation assay

Matrigel (BD Biosciences) was melted at 4°C, added to 48-multiwell plates (Corning) at 100 μl/well, and then incubated at 37°C for 30 min. EPCs (2×10^4^ cells) were resuspended in a 1:1 mixture of MV2 serum-free medium and ATDC5 CM (total 200 μl), and added to the wells. After 12 h of incubation at 37°C, EPC tube formation was assessed by microscopy, and each well was photographed under a light microscope. The numbers of branches were calculated and quantified using MacBiophotonics ImageJ software (NIH).

### ELISA

To measure FGF-2 production of ATDC5 cells in the CM, the cells were seeded into six-multiwell plates (6×10^4^ cells/well) and grown until reaching 80% confluence. Then, they were treated with IL-1β (0–10 ng/ml), and incubated in a humidified incubator at 37°C for different periods of time (0–36 h). To examine the downstream signalling pathways involved in the actions of IL-1β, the cells were pre-treated with various inhibitors for 30 min before 10 ng/ml IL-1β was added. After incubation, the supernatant CM was collected and stored at −80°C until the assay was performed. FGF-2 in the CM was assayed using a human FGF basic DuoSet ELISA Development Kit (R&D Systems) according to the manufacturer's procedure.

### Measurement of ROS

The levels of intracellular H_2_O_2_ were assessed spectrofluorimetrically by the oxidation of a specific probe, H_2_DCFDA (2′,7′-dichlorofluorescin diacetate) (Sigma–Aldrich). Cells were plated at a density of 6×10^4^ and were exposed to 10 ng/ml IL-1β for different time intervals. The cells were stained with 10 mM H_2_DCFDA for 10 min at 37°C, and the fluorescent intensity of cells was determined using a FACSCanto™ flow cytometer (BD Biosciences).

### Transfection and reporter gene assay

ATDC5 cells were grown to 80% confluence in 12-multiwell plates, then co-transfected with 0.8 μg of NF-κB luciferase plasmid and 0.4 μg β-galactosidase expression vector using FuGENE® 6 Transfection Reagent (Promega). DNA and FuGENE were pre-mixed for 15 min and then added to the cells. After 16 h of transfection, the medium was replaced and the cells were incubated under different conditions (with or without IL-1β treatment) for another 24 h. Before performing the reporter assay, the medium was removed, and the cells were washed with ice-cold PBS. Reporter lysis buffer (100 μl) (Promega) was added to each well, and cells were scraped and transferred to a microcentrifuge tube. After a brief centrifugation, the supernatant was transferred to a new tube and the concentration of protein was quantified. Before measuring luciferase intensity, 20 μl of cell lysate was mixed with 100 μl of Luciferase Assay Reagent (Promega) in white opaque 96-multiwell plates. Luminescence was measured using a Fluoroskan™ Ascent FL fluorometer (Thermo Labsystems). Luciferase activity was normalized to the transfection efficiency, which was determined from the activity of the co-transfected β-galactosidase expression vector.

### Western blot analysis

ATDC5 cells were plated on six-multiwell plates and incubated with IL-1β under different conditions, after which cells were lysed with RIPA buffer for 30 min at 4°C. Proteins were separated by SDS/PAGE and transferred to immobile PVDF membranes (Millipore). The membranes were blocked in TBS-T (TBS/0.05% Tween 20) containing 5% (w/v) dried non-fat skimmed milk powder for 1 h at room temperature, and then washed three times. The membranes were incubated with primary antibody (rabbit antibodies against mouse AMPKα1/2, p38α, p65, p-p65, p-AMPKα or FGF-2; or mouse antibodies against mouse β-actin or p-p38) overnight at 4°C. After three washes, the blots were incubated with HRP-conjugated rabbit or mouse secondary antibody for 1 h at room temperature. After incubation with the secondary antibody, proteins were graphically quantified by ECL using an ImageQuant LAS 4000 camera (GE Healthcare).

### CIA (collagen-induced arthritis) mouse model

Male C57BL/6J mice 8–10 weeks old were purchased from National Laboratory Animal Center (Taipei, Taiwan). These mice were maintained according to the guidelines established by the Animal Care Committee of China Medical University. The CIA mouse model was established according to a previous protocol [29,41]. Briefly, in the primary immunization with bCII/CFA emulsion, the bCII (100 μg) was dissolved in dilute acetic acid (0.1 M) to the desired concentration (2 mg/ml) and emulsified in 0.25 ml CFA (1:1) then injected intradermally into the base of the tail. At 3 weeks after primary immunization, collagen/IFA emulsion was used to ensure induction of incidence of CIA. The bCII (100 μg) was emulsified in IFA (1:1) and then injected into the hind leg as a booster. The incidence of arthritis can be observed within 6 weeks after primary immunization and, overall, 95% of the mice will develop severe arthritis.

### IHC (immunohistochemistry) staining

Paraffin-embedded sections were prepared, mounted on silane-coated slides, deparaffinized in xylene, rehydrated in a graded alcohol series and washed in deionized water. After antigen retrieval (sections were heated at 95–100°C on a hotplate for 30 min in 10 mM sodium citrate, pH 6.0), intrinsic peroxidase activity was blocked by incubation with 3% H_2_O_2_. Non-specific antibody-binding sites were blocked using 3% BSA in PBS. Sections were then incubated with appropriately diluted primary antibodies specific for mouse CD31, CD34, CD133 or VEGFR2 at 4°C overnight. After three washes with PBS, the secondary antibody (biotin-labelled goat anti-rabbit IgG) was applied for 30 min at room temperature. Staining was detected with DAB (3,3′-diaminobenzidine tetrahydrochloride) and haematoxylin using a Novolink Polymer Detection Kit (Leica Biosystems). Slides were observed under a light microscope. Specimens from CIA mouse model (knee) were also stained with Safranin-O/Fast Green to evaluate cartilage lesion.

### *In vivo* Matrigel plug assay

All studies using animals were carried out in accordance with the declaration of Helsinki and the Guide for the Care and Use of Laboratory Animals as adopted and promulgated by the National Institutes of Health. The Matrigel plug angiogenesis assay was performed as described previously [42]. Four-week-old male nude mice (National Laboratory Animal Center, Taipei, Taiwan) were divided into four groups (*n*=8 for each group), and subcutaneously injected with 0.15 ml of Matrigel containing ATDC5 CM (PBS, control CM, IL-1β-treated CM, or IL-1β-treated CM with FGF-2 NAb). On day 7, the Matrigel plugs were harvested: some were fixed with 3.7% paraformaldehyde for at least 2 days, and then embedded in paraffin and subsequently processed for CD31 IHC staining, whereas others were evaluated by Drabkin's method (Drabkin's Reagent Kit, Sigma–Aldrich) to quantify the haemoglobin concentration.

### CAM (chick chorioallantoic membrane) assay

*In vivo* angiogenic activity was determined using a CAM assay as described previously [43,44]. Four-day-old fertilized chick embryos were incubated at 37°C in an 80% humidified atmosphere. On developmental day 7, Matrigel containing ATDC5 CM (PBS, control CM, IL-1β-treated CM, or IL-1β-treated CM with FGF-2 NAb) was placed on to the CAMs for 3 more days. The CAMs were then examined by microscopy and photographed. Angiogenesis was quantified by counting the number of blood vessel branches. All animal work was performed in accordance with a protocol approved by the Institutional Animal Care and Use Committee of China Medical University (Taichung, Taiwan).

### Statistical analysis

All statistical analysis and quantitative results were calculated using Prism 6.0 for windows (GraphPad Software) and SPSS 16.0 for windows (SPSS); S.D. are derived from at least three experiments, all parameters were performed the Shapiro–Wilk test to examine the distribution of data; statistical comparison of two groups were performed with Student's *t* test (normally distributed) or Mann–Whitney *U* test (non-normally distributed). In all experiments, the statistical power level is 0.8 and the *n* number of independent experiments is provided in each Figure legend. For all tests, the level of significance was a two-sided *P* value of <0.05.

## RESULTS

### IL-1β induces FGF-2 expression via IL-1RI

**Figure 1:**
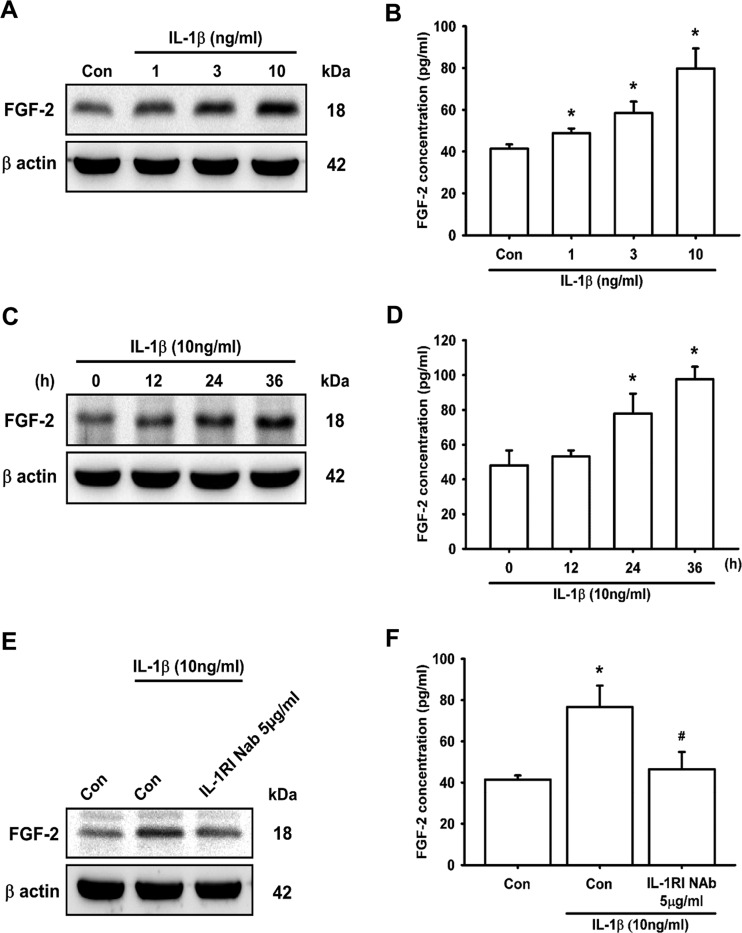
IL-1β induces FGF-2 expression via IL-1RI in chondrocytes ATDC5 cells were incubated with IL-1β in different dosages for 24 h, or with IL-1β for the indicated times. FGF-2 expression was examined by Western blotting (**A** and **C**; *n*≥6) and ELISA (**B** and **D**; *n*≥6). ATDC5 cells were pre-treated with IL-1RI NAb for 30 min, and then stimulated with IL-1β for 24 h, after which FGF-2 expression was examined by Western blotting (**E**; *n*=5) and ELISA (**F**; *n*=6). Quantification results are expressed as means±S.D. *P<0.05 compared with the Con group (control) in (**B**) and (**F**), and compared with the 0 h group in (**D**); #*P*<0.05 compared with the IL-1β-treated group in (**F**). Molecular masses are indicated in kDa. β-Actin was used as a loading control.

Inflammatory cytokine IL-1β has been reported to induce FGF-2 expression in subchondral bone remodelling [45,46] and angiogenesis [46,47]. However, whether IL-1β promotes FGF-2 expression and angiogenesis in chondrocytes is largely unknown. We observed that treatment of chondrocytes (ATDC5 cells) with IL-1β increased FGF-2 protein expression in a concentration- and time-dependent manner (Figures 1A–1D). Moreover, treatment of ATDC5 cells with 10 ng/ml IL-1β for 30 min did not affect the FGF-2 protein expression (Supplementary Figure S2). Previous studies have demonstrated that the IL-1RI plays an important role in inflammatory diseases [48,49] and is associated with IL-1β-induced FGF-2 expression in corneal endothelial cells [50]. We therefore hypothesized that IL-1RI may be involved in IL-1β-induced FGF-2 expression. Pre-treatment with IL-1RI NAb attenuated IL-1β-induced FGF-2 expression (Figures 1E and 1F). Taken together, these results indicate that IL-1β induces FGF-2 expression in chondrocytes through IL-1RI.

### IL-1β promotes FGF-2-dependent EPC angiogenesis *in vitro*

**Figure 2:**
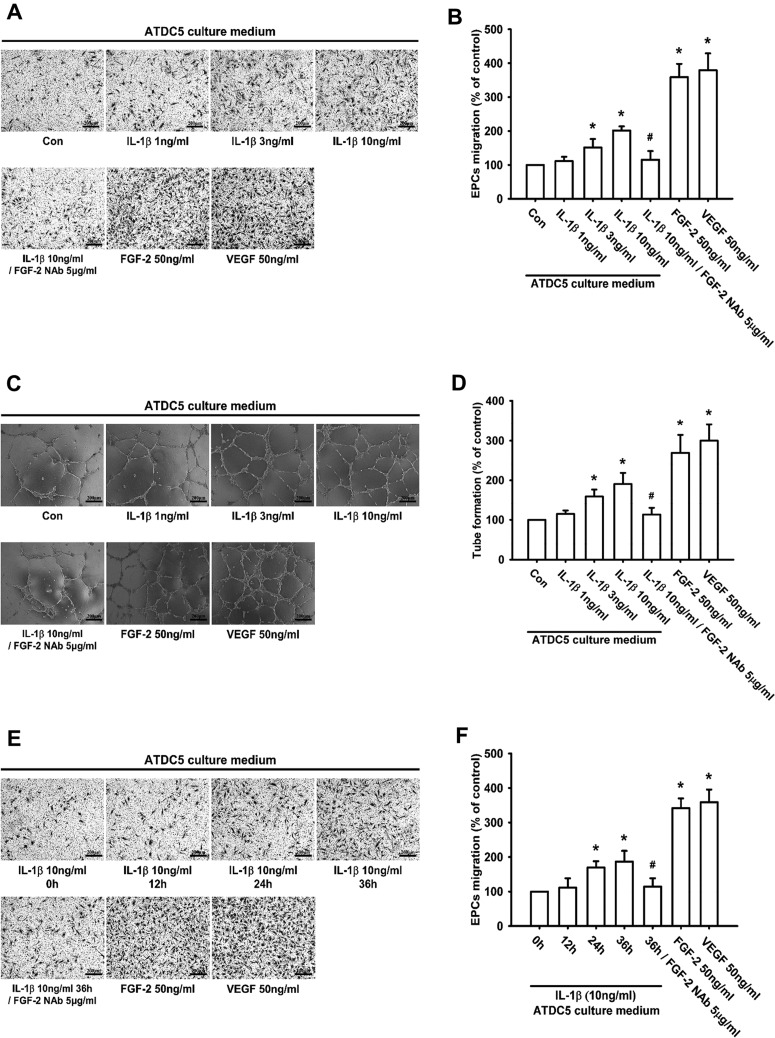
IL-1β-induced FGF-2 expression promotes EPC migration and tube formation ATDC5 cells were incubated with IL-1β at different dosages for 24 h, pre-treated with FGF-2 NAb for 30 min before incubation with IL-1β for 24 h, or incubated with IL-1β for the indicated times. The CM was collected, and then applied to EPCs. An EPC migration assay (**A**, **B**, **E** and **F**; *n*≥6) and tube formation assay were performed (**C** and **D**; *n*=8); Scale bar, 200 μm. Quantification results are expressed as means±S.D. *P<0.05 compared with the Con group (control) in (**B**) and (**D**), and compared with the 0 h group in (**F**); #*P*<0.05 compared with the IL-1β-treated group in (**B**) and (**D**), and compared with the 36 h group in (**F**).

FGF-2 is an important regulator of embryonic vascular development [47]. A previous study indicates that EPCs contribute to angiogenesis during arthritis [29]. We therefore used circulating EPCs to determine whether IL-1β-induced FGF-2 expression promoted angiogenesis *in vitro*. The results demonstrated that EPC angiogenic activities (migration and tube formation) were enhanced by IL-1β treatment in a concentration- and time-dependent manner (Figures 2A–2F). In contrast, pre-treatment of ATDC5 cells with FGF-2 NAb significantly abolished IL-1β-induced EPC migration and tube formation (Figures 2A–2F). Recombinant human FGF-2 and VEGF (vascular endothelial growth factor) were used as positive controls. These results suggest that IL-1β facilitates FGF-2-dependent EPC angiogenesis.

### ROS generation is involved in IL-1β-induced FGF-2 expression

**Figure 3:**
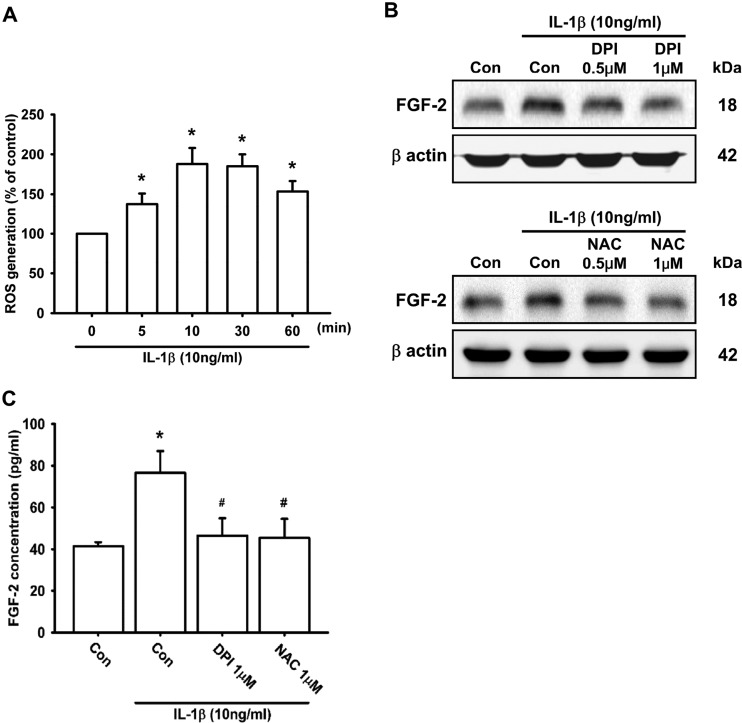
ROS generation is involved in IL-1β-induced FGF-2 expression ATDC5 cells were labelled with 10 μM H_2_DCFDA, and then incubated with IL-1β for the indicated times. The fluorescent intensity was measured by flow cytometry (**A**; *n*=9). ATDC5 cells were pre-treated with DPI or NAC for 30 min, and then stimulated with IL-1β for 24 h. FGF-2 expression was examined by Western blotting (**B**; *n*=8) and ELISA (**C**; *n*=6). Quantification results are expressed as means±S.D. *P<0.05 compared with the 0 min group in (**A**) and compared with the Con group (control) in (**C**); #*P*<0.05 compared with the IL-1β-treated group in (**C**). Molecular masses are indicated in kDa. β-Actin was used as a loading control.

Several studies have shown that ROS generation is required for the IL-1β-mediated signalling pathway [13,51–54]. We therefore used a fluorescent probe to determine whether IL-1β induces ROS production in ATDC5 cells. The results demonstrated that treatment of ATDC5 cells with 10 ng/ml IL-1β increased intracellular ROS levels in a time-dependent manner (0–60 min), and reached its maximum level in 10 min (Figure 3A). However, after 24 h of incubation with IL-1β, the level of ROS was not changed (Supplementary Figure S3). To determine whether ROS took part in IL-1β-induced FGF-2 expression, NAC (a ROS scavenger) and DPI (a ROS inhibitor) were used. Pre-treatment of ATDC5 cells with the ROS scavenger and inhibitor attenuated IL-1β-induced FGF-2 expression (Figures 3B and 3C). These results suggest that ROS generation is involved in IL-1β-induced FGF-2 production.

### The AMPK-dependent p38 pathway is involved in IL-1β-induced FGF-2 expression

**Figure 4:**
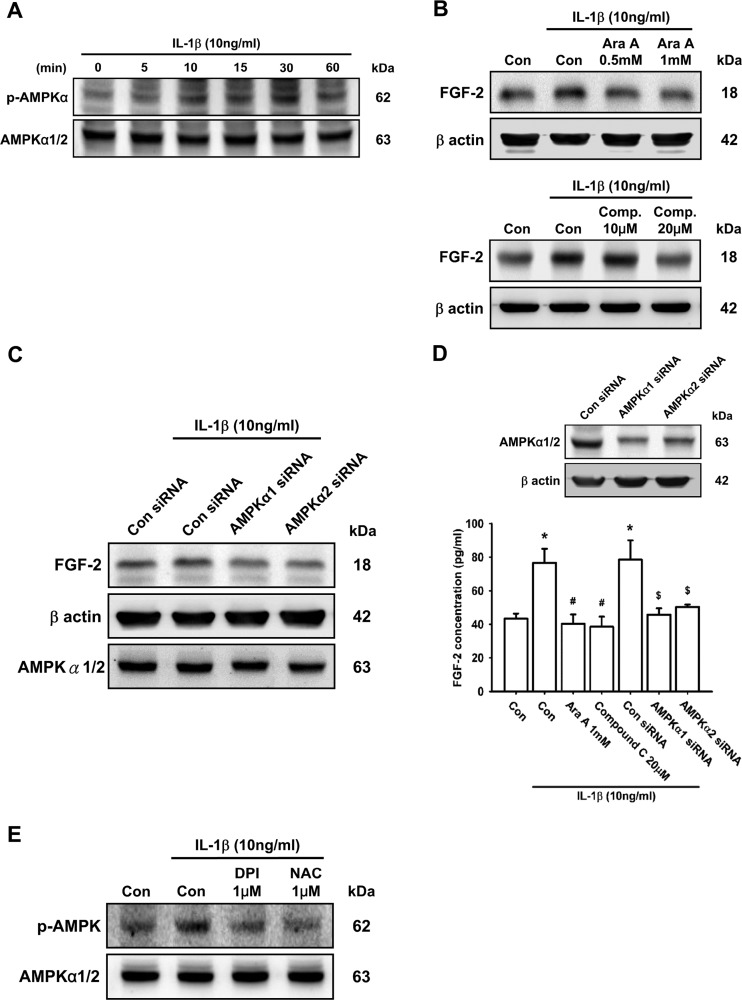
The AMPK pathway is involved in IL-1β-induced FGF-2 expression ATDC5 cells were incubated with IL-1β for the indicated times, and AMPK phosphorylation was examined by Western blotting (**A**; *n*=8). ATDC5 cells were pre-treated with Ara A or Compound C (Comp.) for 30 min or transfected with AMPK siRNAs (α1 and α2) for 12 h, and then stimulated with IL-1β for 24 h. FGF-2 expression was examined by Western blotting (**B** and **C**; *n*≥8) and ELISA (**D**; *n*=6). Cells were pre-treated with DPI or NAC for 30 min, and then stimulated with IL-1β for 15 min, AMPK phosphorylation was examined by Western blotting (**E**; *n*=6). Quantification results are expressed as means±S.D. *P<0.05 compared with the Con group (control); #*P*<0.05 compared with the IL-1β-treated group; $*P*<0.05 compared with the control siRNA-transfected group (**D**). Molecular masses are indicated in kDa. β-Actin was used as a loading control.

ROS/AMPK activation has been reported to improve angiogenesis in pulmonary artery endothelial cells [55]. To examine whether the AMPK signalling pathway was involved in IL-1β-induced FGF-2 production, we measured the effect of time on the phosphorylation level of AMPK. The results revealed that IL-1β enhanced AMPK phosphorylation in a time-dependent manner (Figure 4A). Pre-treatment of ATDC5 cells with AMPK inhibitors, Ara A (upper panel) or Compound C (lower panel), attenuated IL-1β-induced FGF-2 expression in a concentration-dependent manner (Figure 4B). Moreover, pre-transfection with AMPK siRNA (α1 or α2) also decreased IL-1β-induced FGF-2 expression (Figures 4C–4D). On the other hand, pre-treatment of ATDC5 cells with DPI and NAC significantly decreased IL-1β-enhanced AMPK phosphorylation (Figure 4E), indicating that the ROS/AMPK pathway is required for IL-1β-induced FGF-2 expression in chondrocytes.

**Figure 5:**
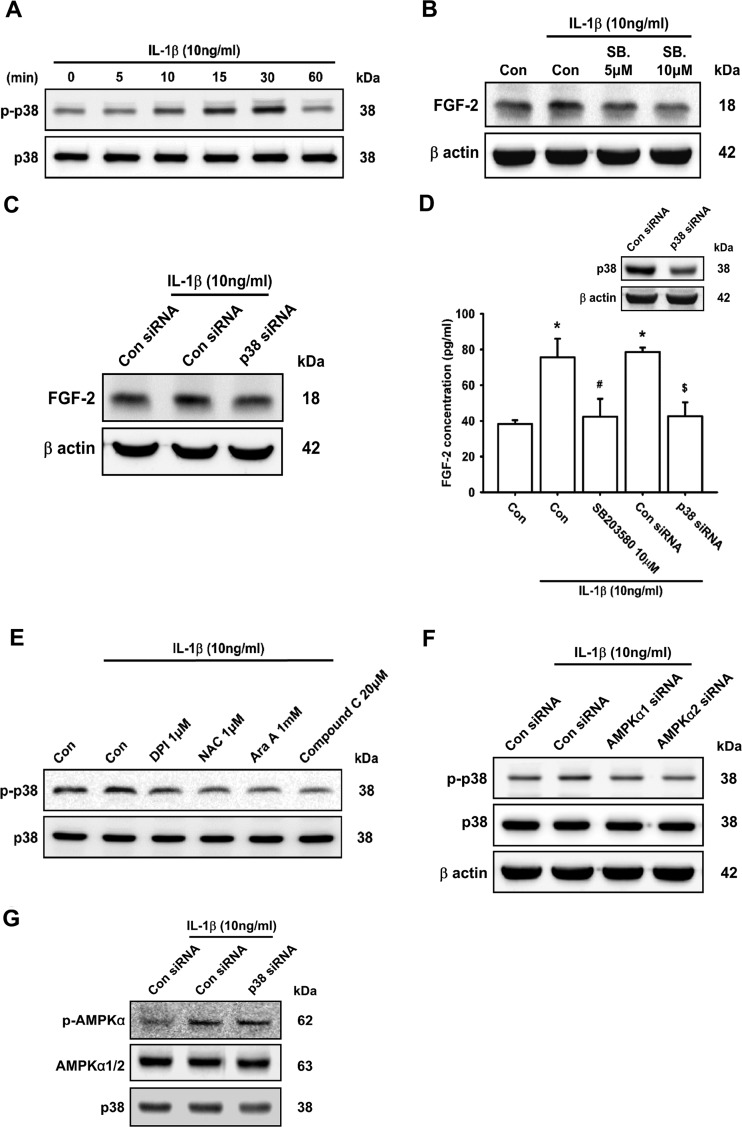
AMPK-dependent p38 activation is involved in IL-1β-induced FGF-2 expression ATDC5 cells were incubated with IL-1β for the indicated times, and p38 phosphorylation was examined by Western blotting (**A**; *n*=7). ATDC5 cells were pre-treated with SB203580 (SB.) or cells were transfected with p38 siRNA for 12 h, and then stimulated with IL-1β for 24 h. FGF-2 expression was examined by Western blotting (**B** and **C**; *n*≥7) and ELISA (**D**; *n*=6). ATDC5 cells were pre-treated with DPI, NAC, Ara A or Compound C for 30 min or cells transfected with AMPK (α1 and α2) and p38 siRNA for 16 h, and then stimulated with IL-1β for 30 min. Phosphorylation of p38 (**E** and **F**; *n*≥8) and AMPK (**G**; *n*=6) was examined by Western blotting. Quantification results are expressed as means±S.D. *P<0.05 compared with the Con group (control); #*P*<0.05 compared with the IL-1β-treated group; $*P*<0.05 compared with the control siRNA-transfected group (**D**). Molecular masses are indicated in kDa. β-Actin was used as a loading control.

A previous study demonstrated that IL-1β-induced FGF expression activated further the p38 pathway and promoted the migration of human corneal endothelial cells [56]. To evaluate whether the p38 signal plays a role in IL-1β-induced FGF-2 expression, p38 phosphorylation was examined. In the present study, we found that treatment of cells with 10 ng/ml IL-1β enhanced p38 phosphorylation in a time-dependent manner (Figure 5A). However, after 24 h of incubation with IL-1β, the phosphorylation of p38 was not changed (Supplementary Figure S4). Moreover, IL-1β-induced FGF-2 expression was attenuated by pre-treatment with p38 inhibitor (SB203580) or cells transfected with p38 siRNA (Figures 5B–5D). Several studies have demonstrated that IL-1β-mediated cartilage degradation occurs via the AMPK/p38 signalling pathway [57,58]. Treatment of ATDC5 cells with DPI, NAC, Ara A and Compound C or cells transfected with AMPK siRNA abolished IL-1β-induced p38 phosphorylation (Figures 5E and 5F). However, p38 siRNA did not have any effect on IL-1β-induced AMPK phosphorylation (Figure 5G), suggesting that the ROS and AMPK-dependent p38 pathway is involved in IL-1β-induced FGF-2 expression in chondrocytes.

### The transcription factor NF-κB is involved in IL-1β-induced FGF-2 expression

**Figure 6:**
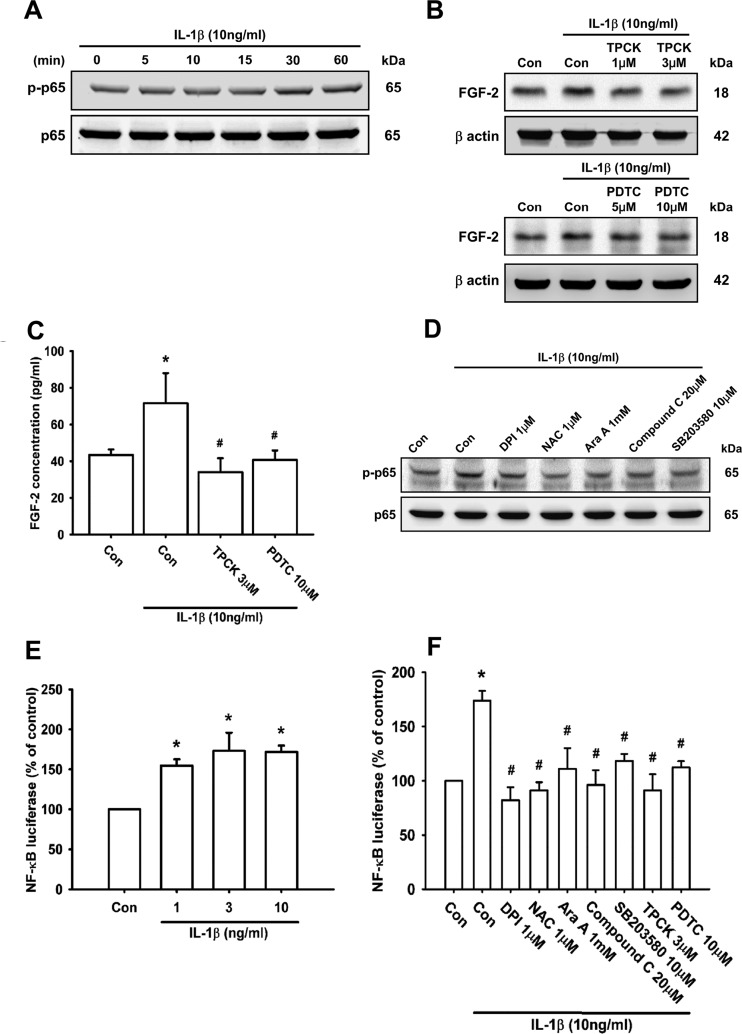
The ROS/AMPK/p38 pathway is involved in IL-1β-induced NF-κB activation and FGF-2 expression ATDC5 cells were incubated with IL-1β for the indicated times, and p65 phosphorylation was examined by Western blotting (**A**; *n*=8). ATDC5 cells were pre-treated with TPCK or PDTC for 30 min, and then stimulated with IL-1β for 24 h. FGF-2 expression was examined by Western blotting (**B**; *n*=8) and ELISA (**C**; *n*=6). ATDC5 cells were pre-treated with DPI, NAC, Ara A, Compound C or SB203580 for 30 min, and then stimulated with IL-1β for 30 min, after which NF-κB (p65) phosphorylation was examined by Western blotting (**D**; *n*=8). ATDC5 cells were incubated with IL-1β at different dosages for 24 h, or pre-treated with DPI, NAC, Ara A, Compound C, SB203580, TPCK or PDTC for 30 min before stimulation with IL-1β for 24 h. NF-κB luciferase activity was measured (**E** and **F**; *n*≥8). Quantification results are expressed as means±S.D. *P<0.05 compared with the Con group; #*P*<0.05 compared with the IL-1β-treated group in (**F**). Molecular masses are indicated in kDa. β-Actin was used as a loading control.

Numerous studies have indicated that NF-κB can be activated by IL-1β [12,59,60], which subsequently induces FGF-2 production [50,56]. First, we examined p65 phosphorylation following IL-1β stimulation. The results demonstrated that treatment of ATDC5 cells with 10 ng/ml IL-1β increased p65 phosphorylation in a time-dependent manner (Figure 6A). Next, pre-treatment of cells with TPCK, an IκB (inhibitor of NF-κB) protease inhibitor, and PDTC, an NF-κB inhibitor, both decreased IL-1β-induced FGF-2 expression (Figures 6B and 6C). To determine whether p65 is a downstream effector of the ROS/AMPK/p38 pathway, we tested inhibitors of ROS, AMPK and p38, and found that they diminished IL-1β-induced p65 phosphorylation (Figure 6D).

In addition, we performed transit transfection with the NF-κB promoter–luciferase construct to confirm that the NF-κB element is involved in IL-1β-induced FGF-2 expression. The activity of NF-κB increased after IL-1β treatment in a concentration-dependent manner (Figure 6E), and significantly decreased following treatment of cells with DPI, NAC, Ara A, Compound C or SB203580 (Figure 6F). On the basis of these results, we conclude that the activation of the ROS, AMPK, p38 and NF-κB pathway is required for IL-1β-induced FGF-2 expression in chondrocytes.

### IL-1β-induced FGF-2 expression enhances angiogenesis *in vivo*

**Figure 7:**
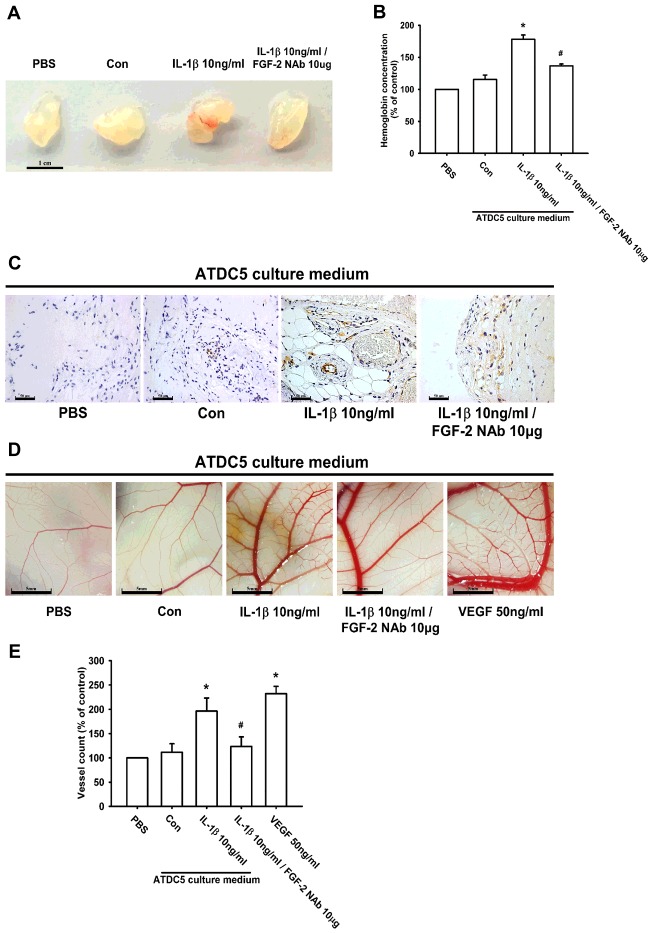
IL-1β-induced FGF-2 promotes angiogenesis *in vivo* Matrigel plugs containing PBS, ATDC5 CM, IL-1β-treated ATDC5 CM or IL-1β-treated CM with FGF-2 NAb were subcutaneously injected into nude mice. After 7 days, the plugs were retrieved and photographed. Scale bar, 1 cm (**A**; *n*=6). Haemoglobin levels were quantified and normalized to the PBS group (**B**; *n*=6). Paraffin sections of Matrigel plugs were stained with CD31 by IHC staining and photographed under a microscope. Scale bar, 50 μm (**C**; *n*=5). The CAM assay utilized 5-day-old fertilized chick embryos. Matrigel was mixed with PBS, ATDC5 CM (Con), IL-1β-treated ATDC5 CM and IL-1β-treated ATDC5 CM with FGF-2 NAb, and then placed on to the CAMs for 3 days. The CAMs were then examined by microscopy and photographed. Scale bar, 5 mm (**D**; *n*=6). Vessels on the CAMs were quantified (**E**; *n*=6). VEGF was used as a positive control in (**D**) and (**E**). Quantification results are expressed as means±S.D. *P<0.05 compared with the PBS group; #*P*<0.05 compared with the IL-1β-treated CM group in (**B**) and (**E**).

Previous studies have confirmed that FGF-2 plays an important role in angiogenesis [61,62]. In order to characterize the angiogenic function of IL-1β-induced FGF-2 expression in chondrocytes, the *in vivo* Matrigel plug assay was used. The plugs of the IL-1β-treated group exhibited a notable increase in blood vessel growth (Figure 7A). Vascular formation in the Matrigel plugs was increased as determined using a haemoglobin content assay, and IHC staining for the platelet endothelial cell marker CD31 confirmed that IL-1β enhanced angiogenesis *in vivo* (Figures 7B and 7C). In contrast, pre-treatment with 10 μg/ml FGF-2 NAb abolished IL-1β-mediated blood vessel growth (Figures 7A–7C). Additionally, in the CAM assay, the IL-1β-treated group also had significantly more capillaries and greater blood vessel formation than did the PBS-treated group (Figures 7D and 7E). IL*-*1β-mediated blood vessel formation was abolished by treatment with FGF-2 NAb (Figure 7E). Therefore IL-1β-induced FGF-2 expression in chondrocytes subsequently promotes angiogenesis *in vivo*.

### CIA increased IL-1β and FGF-2 expression, as well as EPC recruitment *in vivo*

**Figure 8:**
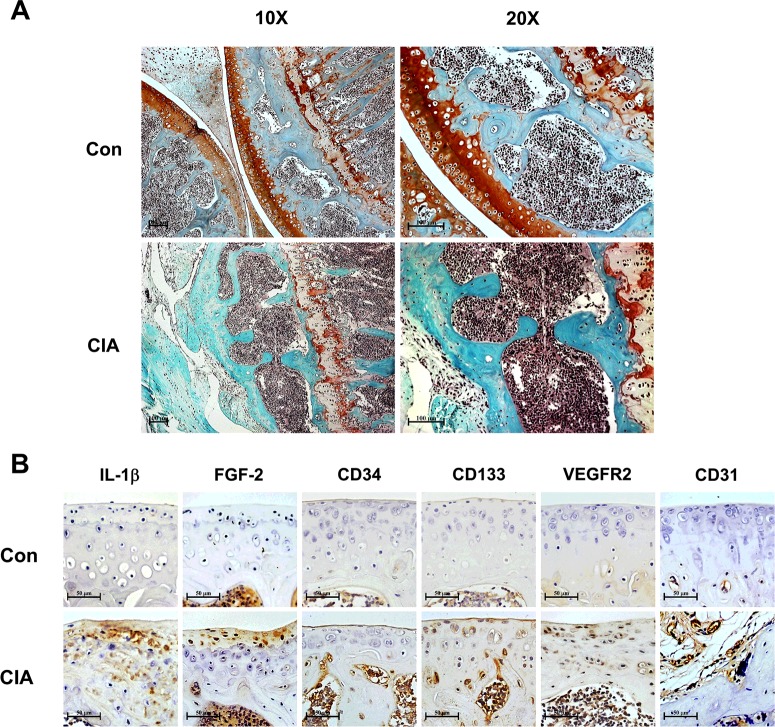
The CIA mouse model increases IL-1β and FGF-2 expression, as well as EPC homing in arthritic joints Paraffin sections of knee joint from control (Con) and CIA mice were stained with Safranin-O/Fast Green. Scale bar, 100 μm (**A**; *n*=4). Articular cartilage was IHC-stained with anti-IL-1β, anti-FGF-2, anti-CD34, anti-CD133, anti-VEGFR2 and anti-CD31 antibodies. Scale bar, 50 μm (**B**; *n*≥5).

To address the EPC recruitment during arthritis, the CIA mouse model was used. Compared with control mice, we found that CIA mice had significant articular cartilage erosion and destruction of the knee joint by Safranin-O/Fast Green staining (Figure 8A). Moreover, CIA significantly induced expression of IL-1β, FGF-2, EPC markers (CD34, CD133 and VEGFR2) and vessel marker (CD31) compared with control mice (Figure 8B). These data suggest that the expression of IL-1β and FGF-2, as well as EPC recruitment, is involved in arthritic development *in vivo*.

## DISCUSSION

Chondrocytes are the only cellular components of cartilage, and they maintain equilibrium between anabolic and catabolic activities. The ATDC5 cell line is characterized as a chondrogenic cell line that goes through a sequential process analogous to chondrocyte differentiation, making it an appropriate *in vitro* model to study the factors that influence cell behaviour. In the present study, we utilized the chondrocytic ATDC5 cell line to examine the role of IL-1β, the major inflammatory cytokine during arthritis, in the expression of the pro-angiogenesis factor FGF-2. Our results suggest that IL-1β promotes FGF-2 expression in chondrocytes through the ROS, AMPK, p38 and NF-κB signalling pathway, and subsequently increases EPC angiogenesis.

Since EPCs were first described a decade ago, evidence has emerged of their contribution to angiogenesis during the pathogenesis of arthritis [17]. Angiogenesis has recently become a possible therapeutic target for patients with the disease [63]. IL-1β is an important cytokine involved in arthritic inflammation [4,64], which leads to progressive cartilage breakdown, disturbed chondrocyte function and matrix degradation [60,65]. However, it is not yet clear whether circulating EPCs are associated with the regulation and mechanistic function of IL-1β during arthritis. EPC migration and tube formation are essential for angiogenesis and important in new vessel formation [29,66]. Our data demonstrate that IL-1β-treated ATDC5 CM facilitated EPC migration and capillary-like structure formation. In addition, FGF-2 NAb diminished IL-1β-mediated EPC migration and tube formation *in vitro*, as well as blood vessel formation in the Matrigel plug assay and CAM model *in vivo*. In the CIA mouse model, we found EPCs homing to articular cartilage. However, EPCs in arthritis were considered to be homing from the circulatory system, or might be differentiated from the chondroprogenitor in articular cartilage. In the present study, we used circulating EPC markers (CD34, CD133 and VEGFR2) [26,27,67], which are negative for the chondroprogenitor [68], to identify the EPCs in CIA mouse joint. Our data demonstrate that the expression of circulating EPC markers was highly increased in CIA mice, suggesting EPC homing from the circulatory system.

In clinical samples, the normal FGF-2 concentration in synovial fluid is ∼10 pg/ml [69]. However, in pathological conditions, the FGF-2 concentration is ∼8.4 (mild)–70.3 (severe) pg/ml in RA patients [23] and ∼3.6 (mild)–57.4 (severe) pg/ml in OA patients [70]. In the present study, IL-1β (10 ng/ml) promoted 80 pg/ml FGF-2 expression in chondrocytes; this result is consistent with severe arthritis patients in the clinic.

IL-1RI, the IL-1β receptor that mediates the biological effects of IL-1β, has been identified [38,64]. Hence targeting IL-1RI with a selective antagonist may provide therapeutic benefits in IL-1β-mediated inflammatory diseases [7,48]. In the present study, we demonstrated that IL-1β-induced FGF-2 expression was attenuated by IL-1RI NAb treatment, suggesting that IL-1β promoted FGF-2 expression in chondrocyte cells through interaction with IL-1RI. Oxidative stress is a major factor in the development and the progression of many pathological conditions, including arthritis [71]. Previous studies have demonstrated that IL-1β induces ROS production in human chondrocytes [51]. Our results revealed that IL-1β in chondrocyte cells stimulated the cellular production of ROS. In addition, IL-1β*-*induced FGF*-*2 expression was attenuated by pre-treatment with NAC and DPI, a ROS scavenger and ROS inhibitor respectively. These results indicate that the expression of FGF*-*2 in chondrocytes induced by IL-1β is involved in the generation of ROS.

AMPK is a heterotrimeric serine/threonine kinase [72]. A previous study has shown that the AMPK/p38 signalling pathway also takes part in ROS-mediated catabolism in muscle cells [73]. In the present study, AMPK inhibitors Ara A and Compound C blocked IL-1β-induced FGF-2 expression, indicating that AMPK activation plays a key role in IL-1β-mediated angiogenesis in chondrocytes. To determine the catalytic subunit of AMPK that mediates IL-1β signalling, we used siRNA against AMPKα1 or AMPKα2, and found that both siRNAs abolished IL-1β-induced FGF-2 expression. This implies that AMPKα1 and AMPKα2 are involved in IL-1β-induced FGF-2 expression and angiogenesis. AMPK-dependent p38 activation has been reported to be involved in angiogenesis [74]. In the present study, we demonstrate that a p38 inhibitor or siRNA decreased IL-1β-induced FGF-2 production. On the other hand, the increased p38 phosphorylation induced by IL-1β in chondrocytes was diminished by the AMPK inhibitors Ara A and Compound C. In contrast, p38 siRNA did not affect IL-1β-induced AMPK phosphorylation, indicating the involvement of AMPK-dependent p38 activation in IL-1β-induced FGF-2 expression and angiogenesis in chondrocytes.

Inflammation is intimately linked to angiogenesis, which plays an important role during arthritis. Thus the development of an anti-angiogenic therapy could conceivably be useful for these patients. In the present study, we found that IL-1β, a key inflammatory cytokine during arthritis, induces the expression of the angiogenic factor FGF-2 in chondrocytes, and subsequently promotes EPC migration and tube formation through the activation of the IL-1RI/ROS/AMPK/p38/NF-κB signalling pathway. The present study provides a better understanding of the mechanisms underlying arthritis pathogenesis, and opens a new window in the search for potential therapeutic targets of arthritis.

### Limitations

Several limitations of the study must be noted. First, we did not have clinical arthritis samples to evaluate the expression of IL-1β and FGF-2, as well as EPC recruitment in articular cartilage. Therefore the clinical relevance may need further examination. Secondly, the ATDC5 cell line is known as a chondrocyte-like cell line derived from mice. Whether naïve human chondrocytes have similar effects needs further confirmation.

## Abbreviations

AMPK: AMP-activated protein kinase
Ara A: adenine 9-β-D-arabinofuranoside
bCII: bovine type II collagen
CAM: chick chorioallantoic membrane
CFA: complete Freund's adjuvant
CIA: collagen-induced arthritis
CM: culture medium
DPI: diphenyleneiodonium chloride
EPC: endothelial progenitor cell
FGF: fibroblast growth factor
H_2_DCFDA: 2′,7′-dichlorofluorescin diacetate
HRP: horseradish peroxidase
IFA: incomplete Freund's adjuvant
IHC: immunohistochemistry
IL: interleukin
IL-1RI: type-1 IL-1 receptor
NAb: neutralizing antibody
NAC: *N*-acetyl-L-cysteine
NF-κB: nuclear factor κB
OA: osteoarthritis
PDTC: ammonium pyrrolidinedithiocarbamate
RA: rheumatoid arthritis
ROS: reactive oxygen species
TPCK: *N*-p-tosyl-L-phenylalanine chloromethylketone
VEGF: vascular endothelial growth factor
VEGFR2: VEGF receptor 2
